# Co-adapting a school-based mental health literacy intervention for adolescents in Cambodia and Vietnam

**DOI:** 10.1136/bmjgh-2026-024547

**Published:** 2026-09-25

**Authors:** Aisling Murray, Nashwa Mohammed, Vân Vũ, Nil Ean, Sovandara Kao, Sareth Khann, Đào Kieu, Mengleang Kong, Khang Lê Hữu Minh, Phuong Hong Ngoc Nguyen, Phuong Nguyen, Bunna Phoeun, Thearom Ret, Phuong Tran Thi Mai, Francois van Loggerenberg, Channith Yeun, Hoang-Minh Dang, Fiona Samuels

**Affiliations:** 1 Global Public Health Unit, Centre for Public Health and Policy, Wolfson Institute of Population Health, Queen Mary University of London Faculty of Medicine and Dentistry, London, UK; 2 Youth Resilience Unit, Centre for Psychiatry and Mental Health, Wolfson Institute of Population Health, Queen Mary University of London Faculty of Medicine and Dentistry, London, UK; 3 University of Education, Vietnam National University Hanoi, Hanoi, Viet Nam; 4 The Center for Trauma Care and Research Organization (CTRO), Phnom Penh, Cambodia; 5 Department of Psychology, Royal University of Phnom Penh, Phnom Penh, Cambodia; 6 Department of Organizing Event and Sports Statistics, Royal Government of Cambodia Ministry of Education Youth and Sport, Phnom Penh, Cambodia

**Keywords:** Global Health, Mental Health & Psychiatry, Other study design

## Abstract

**Introduction:**

Given rising rates of mental health difficulties among young people, early intervention is crucial to mitigate personal and societal costs. Interventions should be locally adapted to ensure acceptability and sustainability, particularly in low- and middle-income countries (LMICs), where health systems are often under-resourced. Vietnam and Cambodia are LMICs with young populations, recent histories of conflict and ongoing vulnerabilities, including poverty and urban-rural inequality. While many adolescents experience common mental disorders, access to care is limited by stigma, low mental health literacy and reliance on urban, medicalised services. Community-based interventions are therefore needed; however, evidence on adapting such interventions in LMICs remains limited. This article describes the co-adaptation of a school-based mental health literacy intervention in Vietnam and Cambodia.

**Methods:**

The intervention, within the Mental health capacity Building and stRengthening In Global HealTh systems study, was adapted from the co-created Adolescent Mental Health Promotion intervention implemented in Vietnam and Tanzania. Adaptations to content and delivery were developed through workshops across four study sites (two in each country). Facilitated by local research teams, the workshops engaged adolescents alongside parents, teachers and other stakeholders. Activities included topic ranking, workbook exercises and group discussions. Adaptations were synthesised across sites using spreadsheets, and feedback from stakeholder advisory groups informed the final intervention design.

**Results:**

Key adaptations included delivery by two trained facilitators per session (one teacher, one external provider, with an additional back-up teacher in Cambodia), inclusion of peer-led co-facilitation of some sessions and the addition of new topics: ‘the Internet’, ‘Eating disorders and body image’ and ‘Self-harm and suicide’.

**Conclusion:**

Co-adaptation was strengthened by early and diverse stakeholder engagement and participatory workshops that enabled all stakeholders, including adolescents, to voice their views. Responsive adaptations will likely continue throughout delivery via participant input, research team discussions and consultations with stakeholder advisory groups.

**WHAT IS ALREADY KNOWN ON THIS TOPIC**

- School-based mental health programmes are a promising strategy to support adolescent mental health. Such interventions are limited in low- and middle-income countries (LMICs) despite their potential to improve access in contexts where formal youth mental health services are scarce.
- Co-adaptation can improve contextual relevance and sustainability of interventions, yet there is limited evidence on co-adaptation processes, including for youth mental health interventions in LMICs.

**WHAT THIS STUDY ADDS**

- Through co-adapting a mental health literacy intervention in Vietnam and Cambodia, this study demonstrates that co-adaptation can be successfully completed in LMIC settings where there has been relatively limited prior use of these approaches.
- This study highlights the value of involving diverse stakeholders, including youth, across multiple system levels to bring diverse perspectives and ensure buy-in from key actors who could support intervention sustainability.

**HOW THIS STUDY MIGHT AFFECT RESEARCH, PRACTICE OR POLICY**

- Our findings show that a structured co-adaptation process can be conducted in LMIC settings and can generate locally relevant adaptations while maintaining core intervention functions.
- These findings underscore the need for greater inclusion of LMIC settings in youth mental health intervention development and adaptation.

## Introduction

 Adolescence (10–19 years) is a formative period of physical, emotional and social development.^1^ Mental disorders constitute 15% of the global burden of disease among adolescents,^1^ with approximately 75% of lifetime mental disorders emerging before age 25.^2^ Adolescents comprise nearly one-fifth of Southeast Asia’s population, where the prevalence of mental disorders among this age group is high.^3^ Discrimination, stigma and limited resources are barriers to youth accessing effective and timely care. Additionally, poverty, climate change and humanitarian emergencies pose ongoing and new challenges to mental health.^4^

Vietnam and Cambodia are middle-income countries in Southeast Asia with relatively young populations; 23% and 34% of their populations are aged 0–14 years, respectively.^5 6^Both countries share histories of 19th–20th French colonial rule and 20th century conflicts (ie, the Vietnam War (or American War in Vietnam), the Cambodian Civil War and Cambodian genocide) and ongoing vulnerabilities including poverty and urban-rural inequality.^7 8^ Prevalence of common mental disorders among youth is high; up to 41% of Vietnamese youth experience depression and 23% anxiety,^9^ while in Cambodia rates of depression, anxiety and post-traumatic stress disorder are 17%, 27% and 8%, respectively.^10^ Mental health outcomes are also influenced by intergenerational trauma,^11–13^ low mental health literacy, stigma^14–17^ and shortages of mental health workers. Cambodia has approximately 60 psychiatrists (1 per 260 000 people)^16^ and Vietnam around 900 for a population of 100 million.^18^ These shortages reflect the legacy of colonial practices (eg, urbanised and institutionalised treatment of mental illness)^19^ and the impact of 20th century conflicts (eg, trauma and limited resources).^20^

While both countries have implemented community-based mental health services, coverage remains limited. Vietnam’s national community mental healthcare project (2000–2015) delivered care at the commune level, but focused on severe mental disorders and did not target youth.^21^ While school-based psychological counselling units exist, uneven coverage, variability in quality and confidentiality concerns limit uptake.^22^ In Cambodia, government mental health clinics rapidly expanded in the late 1990s with international funding, but had significantly scaled back by 2010.^16^ While non-governmental organisation (NGO) services fill gaps, programmes are often geographically limited, time-bound and rarely youth-focused.^16^ Strengthening community-level, youth-focused services is therefore a priority in both countries,^9 16 23 24^ and youth mental health is increasingly a national policy priority.^25 26^

Within these contexts, schools represent an opportune setting for promoting adolescent mental health and well-being.^24^ School-based mental health programmes (SBMHPs) may help decentralise services and improve access, particularly in low- and middle-income countries (LMICs) where formal services are scarce.^27^ Evidence from high-income countries suggests that SBMHPs can support students’ emotion regulation, reduce mental health symptoms and improve school belonging,^28–30^ and while evidence from LMICs is promising, such interventions remain limited.^31 32^

SBMHPs can be co-created, which involves engaging relevant stakeholders throughout the intervention lifecycle, from problem identification and solution development to implementation and evaluation.^33^ Co-creation includes other participatory approaches such as co-design, co-production, participatory research and Community-Based Participatory Research (CBPR), which aim to create context-specific interventions through multistakeholder engagement, contextual knowledge production, shared learning and open, trustful and inclusive dialogue.^34^ However, they differ in the timing and extent of stakeholder engagement, such as whether stakeholders are involved in intervention design (eg, co-design) or implementation strategy (eg, co-production), who the stakeholders are (eg, members of a specific community, as in CBPR) and sometimes in what they aim to achieve (eg, social change or specific actions).^34^

A co-created SBMHP developed in one context may not translate directly to others due to differences in culture and health and education system infrastructure. Working with stakeholders to adapt SBMHPs to local contexts is therefore important to improve relevance and uptake. Co-adaptation is a process of collaborative adaptation which applies the core elements of co-creation of mutual learning, shared decision-making and stakeholder engagement to adapt an existing intervention to a new context.^33^ It can be done before the intervention begins (planned adaptations) or during delivery to address emerging barriers or unexpected needs (responsive adaptations).^34^ Co-adaptation helps to maintain the intervention’s core components while increasing contextual relevance and potential effectiveness.^35^

Despite its value, explicit documentation of co-adaptation processes remains under-reported, which may limit researcher confidence in using it. de Boer *et al*’s^33^ rapid review on co-adaptation of public health interventions found that few studies clearly document their co-adaptation processes, including stakeholder engagement approaches and co-adaptation session details. Evidence from LMICs is particularly limited, especially for youth mental health interventions.^36^ Increased reporting and utilisation of co-adaptation could improve understanding of how interventions can be effectively adapted across diverse contexts, in turn strengthening mental health systems in LMICs.^37^

This article describes the co-adaptation of a school-based mental health literacy intervention in Vietnam and Cambodia. It aims to (1) document the process of co-adaptation used in the Mental health capacity Building and stRengthening In Global HealTh systems (M-BRIGHT) study as an example of how co-adaptation can be done in LMICs and comparable cultural contexts and (2) discuss challenges and opportunities related to co-adaptation.

## Methods

### Original Adolescent Mental Health Promotion intervention

The M-BRIGHT intervention is a direct adaptation of the school-based Adolescent Mental Health Promotion (AMP)^38^ intervention, co-created with adolescents, parents, teachers and local authority representatives in Tanzania and Vietnam to support adolescent mental health and well-being.^39–41^ Co-creation included 3-day workshops in schools in both countries, which were facilitated by local research teams and involved presentations, small group activities and plenary discussions.

The resulting intervention was a mental health literacy programme, delivered in schools by teachers (Tanzania) or local psychologists (Vietnam) with adolescent support. In Vietnam, on which the M-BRIGHT intervention was based given the comparable Southeast Asian context, the ‘Psychology clubs’ ran twice monthly over 11 months to groups of approximately 30 adolescents and were delivered outside of school hours (20 sessions of approximately 90 min, alternating between indoor and outdoor formats). Sessions built mental health literacy through lectures, group discussions and Q&As (indoor) and practical activities to apply these learnings and strengthen coping skills (outdoor). Topics included: (1) *Getting to know you*, (2) *Overview of mental health*, (3) *Stress*, (4) *Anxiety*, (5) *Depression*, (6) *Emotional regulation*, (7) *Social relationships with parents*, (8) *Social relationships with peers*, (9) *School violence and coping strategies*, (10) *Self-care* (11) *Summary*.

The AMP intervention demonstrated positive effects on adolescent mental health, mental health literacy and coping strategies, and schools and local authorities expressed interest in sustaining and scaling up the intervention.^41^ M-BRIGHT was conceptualised by the AMP co-principal investigators (co-PIs; H-MD, FS) in collaboration with Cambodian researchers, selected given its comparable Southeast Asian context and to build on established relationships between the Vietnam and Cambodia research teams. Equity and research capacity building are core principles of M-BRIGHT, operationalised through local research teams playing central roles in project management and conducting research and South-South learning whereby the Vietnam team shares learnings from AMP with their Cambodian counterparts.

### M-BRIGHT study setting

The M-BRIGHT study includes four sites: Kampong Chhnang and Siem Reap in Cambodia, and Khánh Hòa and Thái Bình in Vietnam. These sites were selected to reflect the diversity of each country’s geography and culture, and as secondary cities with sparser mental health services than country capitals. Seven schools in each country will participate: four schools will be in the ‘urban’ sites (Khánh Hòa and Siem Reap) and three in ‘rural’ sites (Thái Bình and Kampong Chhnang), with one control school in each site. This article details the M-BRIGHT co-adaptation process; full details of site selection, recruitment and intervention testing are reported in the intervention phase protocol.^42^

### Co-adaptation process

We adapted the AMP intervention for a cluster-randomised controlled feasibility trial in Cambodia and Vietnam to test the feasibility, acceptability, fidelity and potential outcomes of the adapted intervention. The longer-term goal is to embed and scale up the intervention,^42^ which was also identified as a critical next step in the AMP study. The study includes four phases: (1) stakeholder analysis (SA) and mapping, (2) training and co-adaptation, (3) intervention testing and (4) embedding and scaling up the intervention; this phase spans the entire project lifespan given its focus on intervention sustainability. This article details the co-adaptation process. Given that adaptation frameworks have been described as overly prescriptive, offering guidance on what is optimal rather than feasible in practice,^43^ we developed a four-pronged approach to ensure adaptability and flexibility of our approach across countries. Thus, co-adaptation was informed by (1) the co-creation workshop design from the AMP study, (2) research team expertise, including members involved in AMP and those new to this study with country-specific knowledge or participatory research experience, (3) the ADAPT guidance^35^ (described below) and (4) recommendations for co-adaptation from researchers with experience, reported in de Boer *et al*.^33^

The ADAPT guidance^35^ provides a systematic, iterative and flexible approach to adapting evidence-based interventions to new contexts.^35^ Developed through systematic review methods, interviews, consultation and formal consensus methods, ADAPT includes step-by-step guidance on assessing rationale for intervention, undertaking adaptations, undertaking piloting and evaluation and implementing the intervention at scale. We particularly drew from its emphasis on early and continuous stakeholder involvement and guidance on documenting planned and responsive adaptations. Where relevant, we have specified how this guidance informed co-adaptation. For instance, we involved stakeholders through advisory groups and co-adaptation workshops to inform planned adaptations to content and delivery and established mechanisms to monitor delivery and address emerging needs for responsive adaptations. Stakeholder engagement began during an SA and mapping phase (phase 1) and mental health literacy training prior to co-adaptation (phase 2). As these study phases informed and fed into the co-adaptation process, we have summarised them below. Figure 1 visualises phases 1 and 2 of the study, with a focus on co-adaptation.

**Figure 1:**
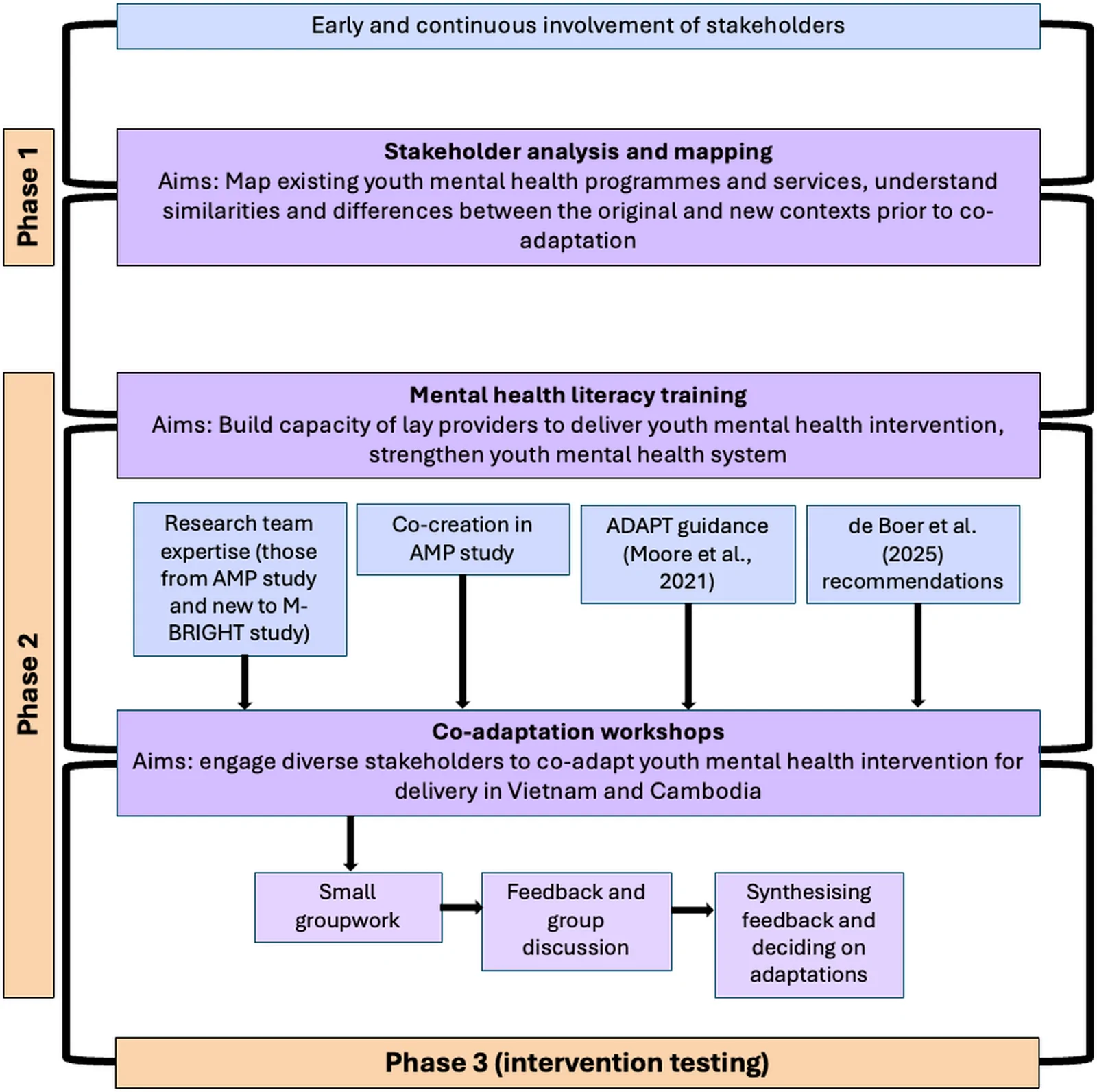
Overview of phases 1 and 2 of the M-BRIGHT study, with a particular focus on the co-adaptation process. Orange boxes indicate the study phases; purple boxes display key study activities; blue boxes show important components which informed co-adaptation. AMP, Adolescent Mental Health Promotion; M-BRIGHT, Mental health capacity Building and stRengthening In Global HealTh systems.

### SA and mapping and mental health literacy training

Prior to co-adaptation, an SA and mapping stage confirmed the study sites and schools and identified: (1) existing mental health programmes and services; (2) potential intervention providers to participate in the mental health literacy training and (3) key decision-makers for in-country stakeholder advisory groups. As per ADAPT guidance, this enabled us to understand similarities and differences between the original and new contexts prior to co-adaptation, including resource availability, delivery systems and workforce capacity. Mapping involved a desk review of secondary and grey literature on existing youth mental health provision in the study sites and relevant national policies, found through existing organisational contacts and online searches. This resulted in a list of stakeholders to contact for key informant interviews to explore these in more depth (21 in Vietnam, 12 in Cambodia from government, unions and NGOs) and consider their involvement in M-BRIGHT going forward. SA workshops were then held in each study site with representatives from NGOs, schools, unions and education, health and social services sectors, the economy sector, healthcare facilities, provincial government (Vietnam only) and religious organisations (Cambodia only) (28 participants in Vietnam, 27 in Cambodia). A key finding was the importance of monks and religious leaders as trusted community figures in Cambodia and the need to involve them in M-BRIGHT training and co-adaptation.

Through the SA, approximately 40 stakeholders across both countries were identified to take part in mental health literacy training, including parents, teachers, community and religious leaders and NGO and governmental stakeholders. Facilitated by the local research teams, this involved training on topics related to youth mental health. (The training is being manualised in Vietnamese, Khmer and English and each version will be made available online.) 20 stakeholders in each country continued their involvement in M-BRIGHT, either as intervention providers or members of the in-country stakeholder advisory groups, selected based on interest and capacity. In Vietnam, there are seven stakeholder advisory group members from WHO, the Ministries of Health and Education, provincial Departments of Education and provincial psychiatric hospitals. In Cambodia, there are 10 members from the Ministry of Education, Youth and Sports, the provincial government, NGOs, a psychiatric hospital and a Monk association. These groups contributed to co-adaption workshops described below and will continue advising on scaling up and embedding the intervention and influencing policy beyond the study lifespan.

The SA and mapping also identified six potential new mental health topics for inclusion in the intervention: *Behavioural disorders*; *Developmental disorders*; *Eating disorders and body image*; *the Internet*; *Self-harm and suicide*; *Substance abuse*. These topics were included in the workshops described below.

### Co-adaptation workshops

Three-day workshops were held in all four study sites in July and August 2025. Consistent with ADAPT guidance,^35^ these aimed to inform changes to intervention content, language, modes of delivery and locations while maintaining the core function of improving mental health literacy and mental health.

Workshop design was led by VV, with input from AM and FS. Content was adapted for Cambodia through an online meeting with VV and the Cambodian team and an in-person meeting with VV, AM, FS and the Cambodian team. Adaptations included translating materials into Khmer, adjusting timings to typical working hours and adding locally relevant icebreakers. Workshops were facilitated by local research teams, including one Community Engagement and Involvement (CEI) Advisor per country, both of whom were psychology students and one of whom was a previous AMP participant. VV supported the Cambodia team in one site (Siem Reap) and UK team members (AM, FS) supported one site in each country (Thái Bình, Siem Reap). In Thái Bình, logistical challenges required condensing the workshop into 1 day; thus, the plenary session was shortened. Learnings from earlier workshops were integrated into subsequent ones—that is, content and structure were tweaked from one workshop to the next, both within and across countries.

#### Participants

Workshops engaged adolescents in grades 10–11 (aged 15–17 years, that is, the target group for the intervention), parents, teachers, community and religious leaders and government and NGO representatives, including stakeholder advisory group members and mental health literacy trainees (see online supplemental file 1 for participant details). All schools involved were participating in the study, and selected teachers were either members of youth unions (ie, in leadership roles), school counsellors or mental health literacy trainees. Adolescents were selected by head teachers for being in leadership positions, perceived to be confident to share their views in groups and representing a mix of genders. Parents were selected for being active school community members who were not parents of participating adolescents. These criteria ensured participants were comfortable representing their school community and contributing to group activities. This was important given that all participants completed the same activities during the workshops. This was done to gather a range of perspectives during group discussions, while small groupwork ensured anyone less confident had their views captured. Total participant numbers were 70 in Cambodia (35 per site) and 67 in Vietnam (29 in Thái Bình, 38 in Khánh Hòa) (online supplemental file 1).

#### Workshop activities

##### Days 1 and 2

Workshops included icebreakers, presentations, small group work and plenary discussions (see online supplemental file 2 for an agenda from Vietnam). Following a ‘speed dating’ icebreaker, the PIs presented a study overview. Next, in small groups (mixed adult groups and separate adolescent groups), participants completed a topic matching activity, whereby cut-outs of topic titles and descriptions from the AMP intervention were provided, and groups were invited to match them up. A researcher then presented the topics from the AMP intervention. These activities familiarised participants with the topics and built confidence working in small groups.

Next, sheets of paper with topic titles were placed around the room and participants were given stickers/sticky notes to vote for the topics they thought were most important for the M-BRIGHT intervention. Following the first workshop in Thái Bình, the *Overview of mental health topic* was removed from voting, given that it received few votes but was deemed essential to the intervention’s core function.^35^ Thus, participants voted across the eight remaining topics in subsequent workshops. Next, an overview of the AMP intervention sessions was presented by a CEI advisor (Vietnam) or VV (Cambodia) to familiarise participants with the intervention structure.

A structured workbook, adapted from the AMP co-creation workbook and using adolescent-friendly language, guided the small groupwork (groups of 4–5, split into adolescent-only and mixed adult groups). The workbook gathered feedback on intervention delivery (eg, providers, format, timing, location, frequency) and topic-specific feedback (eg, content, materials, language). Groups also suggested objectives, activities and materials for three new topic ideas; in three sites (Thái Bình, Kampong Chhnang, Siem Reap), this included *Self-harm and suicide*, *the Internet* and *Behavioural disorders*. These were selected as in the first workshop (in Thái Bình) they received the most votes of the six new topic ideas, so suggestions were sought across the other sites for consistency. In Khánh Hòa, *Eating disorders and body image* was selected instead of *Behavioural disorders* as it received more votes and had been identified as an important, stigmatised topic during the SA and mapping phase. To ensure that suggestions were collected on all topics within the timeframe, different groups provided ideas for different topics, with a minimum of two groups per topic.

##### Day 3

An overview of the feedback was presented back to participants to enable member checking and prompt further discussion and feedback on adapting the intervention.

##### Collating feedback and deciding on adaptations

Workshop discussions were documented through note-taking and workbook and activity outputs. AM, VV and FS created spreadsheets to synthesise participant feedback across sites, with responses translated into English for cross-site comparisons. This included word-for-word translated responses and summaries of responses for each workbook question in each site. Country teams reviewed these and met to decide on key intervention components, guided by consistency of feedback, feasibility within each country and school system and maintenance of key intervention components, including its focus on mental health literacy and well-being. In both countries, online meetings were held with the mental health literacy trainees to gather additional input. Where stakeholder views diverged, decisions were made through research team discussions to maintain feasibility, acceptability and core intervention functions. The draft intervention outline was shared with in-country stakeholder advisory groups for review; in Vietnam, an online meeting was then held, while in Cambodia feedback was gathered through the group’s Telegram chat (Telegram being a common platform for business communication in Cambodia). The Vietnam team prepared the final intervention outline and shared this in English with the Cambodia team. Both teams then created their own detailed session content in local languages and with local adaptations.

### Patient and public involvement

CEI is a core principle of M-BRIGHT, with co-adaptation a key component of this. CEI advisors brought a youth perspective to workshop design and facilitation. Additionally, adolescents, parents, teachers and other stakeholders participated in the workshops and contributed to shaping the intervention. Stakeholder advisory groups will continue to inform intervention delivery, dissemination and scale-up throughout the study.

## Results

The results presented below outline the planned adaptations to the AMP intervention, developed through collation of feedback from the workshops, stakeholder advisory group input and research team discussions. Table 1 provides a side-by-side comparison of the AMP and M-BRIGHT interventions following the development of adaptations for M-BRIGHT.

**Table 1:** An overview of the AMP and M-BRIGHT interventions

Intervention component	AMP	M-BRIGHT
Location	School premises; outside of school hours	School premises; outside of school hours
Length	One topic per month, via two sessions of 90 min each, over 10 months	One topic per month, via two sessions of 90 min each, over **9 months**
Participants	~30 students (10–19 years)	**~35 students (14–18 years)**
Session delivery	Indoor and outdoor sessions	Indoor and outdoor sessions
Intervention providers	Local psychologists (+adolescent peer leader support)	**One teacher** (**+backup in Cambodia**)+**one other trained external provider** (**indoor)**; **peer-led by adolescents** with trained provider supervision (**outdoor**)
Topics	Nine topics	Nine topics; **three new topics: ‘the Internet’, ‘Eating disorders and body image’ and ‘Self-harm and suicide’**

Planned adaptations for M-BRIGHT are highlighted in bold.

AMP, Adolescent Mental Health Promotion; M-BRIGHT, Mental health capacity Building and stRengthening In Global HealTh systems.

### Intervention structure

The final structure of the intervention is as follows: one topic will be covered per month, via two sessions (one indoor, one outdoor) of 90 min each, which will take place on school premises. While some workbook feedback suggested locations such as pagodas, parks and cafes (particularly in Cambodia), most feedback suggested school premises such as school halls and classrooms. The inclusion of indoor and outdoor sessions at school was therefore maintained from the AMP study, as feedback from teachers and adolescents across sites suggested that this would be feasible at their schools and logistically easier for delivery. While some adolescents suggested delivering the intervention during lessons (eg, English or Home Economics, as suggested by a group of adolescents in Kampong Chhnang), the consensus was for it to be delivered as an extracurricular activity outside of class time, which also aligned with school preferences to avoid interrupting students’ normal curriculum.

### Intervention providers

In the M-BRIGHT intervention, indoor sessions will be facilitated by M-BRIGHT trained adult providers, while outdoor sessions will be peer-led by adolescents, with supervision from adult providers. The providers will include one teacher and one external provider. External providers include psychiatrists, psychologists, nurses and youth union leaders (Vietnam) and NGO and social workers (Cambodia) who participated in the mental health literacy training. Providers may rotate leading the sessions. In Cambodia, an additional trained teacher will rotate facilitation with the other teacher. This was based on feedback from teachers during the workshops in Cambodia, whereas this was not deemed feasible in Vietnam due to limited capacity.

This represents an adaptation from the original AMP intervention, in which all sessions in Vietnam were delivered by local psychologists with adolescent peer leader support. For M-BRIGHT, stakeholders suggested involving teachers to ensure sustainability of the intervention beyond the study period, and diverse community-based providers, that is, adults trusted by adolescents within local contexts, to ensure intervention acceptability. The M-BRIGHT intervention also places greater emphasis on peer-led co-facilitation of some sessions as this was suggested to encourage youth ownership.

### Content

The final topics are as follows: (1) *Introduction and overview about mental health* (one indoor session); (2) *Stress and anxiety* (two sessions); (3) *Depression and emotional regulation* (two sessions); (4) *Eating disorders and body image* (two sessions); (5) *Self-harm and suicide* (two sessions); (6) *Social relationships* (with parents and peers) (two sessions); (7) *School violence* (two sessions); (8) *Internet and psychosocial issues among adolescents* (one indoor session); (9) *Self-care and Review* (one outdoor session). As the intervention period is shorter for M-BRIGHT compared with the AMP study (approximately 9 months vs 11 months), some topics were combined.

Stakeholders suggested revising some of the language and examples in the intervention manual to ensure local relevance. As the AMP intervention was co-created in Vietnam, most of these suggested content changes were in Cambodia. For example, feedback from the workbooks suggested simplifications of technical language in the *Depression and emotional regulation*, *Social relationships* and *School violence* topics. An additional suggestion was to include more games and activities in place of lecture-style content.

Three new topics were added to the intervention: *the Internet*; *Eating disorders and body image*; and *Self-harm and suicide*. These were decided through the topic ranking activity (the results of which are shown in online supplemental file 3), feedback from the stakeholder advisory groups after the workshops and local research team clinical expertise. The top two most voted for topics, *the Internet* and *Self-harm and suicide*, were selected to add to the intervention. *Eating disorders and body image* did not receive the top three most votes, possibly due to stigma, limited understanding of eating disorders as a form of mental illness and/or misperceptions that these issues are uncommon. However, the local research teams deemed it important to include based on their clinical and research experience that these are prevalent and stigmatised issues affecting adolescents in both countries. Interestingly, the Cambodia team thought that *Substance abuse* would receive many votes as this is a prevalent issue affecting young people.^44^ However, participants reported that because there are existing programmes related to this in schools, they did not deem it as important for inclusion in the intervention. Stakeholder feedback was incorporated to develop content for the new topics. For example, for *Self-harm and suicide*, feedback from adolescents was incorporated through including definitions of self-harm and suicide and types of self-harm in the indoor session. Similarly, for *Body image and eating disorders*, adolescents suggested including learning and activities on definitions of each, signs and misconceptions of eating disorders and body acceptance. Example content includes a body appreciation activity in the outdoor session.

### Responsive adaptations

As per ADAPT guidance and recommendations for co-adaptation from de Boer *et al*,^33^ responsive adaptations will likely occur throughout delivery via adolescent participant and intervention provider input, research team discussions and consultations with stakeholder advisory groups. This will be informed by an ongoing process evaluation using a spreadsheet to track attendance, fidelity to the intervention, intervention provider feedback and informal feedback from participants following each session. This information will be reported to the wider research team at monthly meetings and any necessary adaptations to improve intervention delivery will be discussed and agreed on. Additionally, midway through the intervention, members of the research team will observe some sessions to monitor fidelity to the intervention and determine whether any adaptations to delivery are needed. Responsive adaptations may include changes to address emerging barriers to delivery and attendance of both the intervention providers and adolescent participants.^45^

Responsive adaptations have already taken place; for example, in one school in Thái Bình, there will be three intervention sessions per month (two indoor, one outdoor), owing to administrative changes within the school and provincial government. This iterative process, involving diverse stakeholders throughout delivery, will follow Albers *et al*’s recommendation for pragmatic fidelity, whereby the core purposes of the intervention are maintained while ensuring it is contextually adapted and acceptable.^46^

## Discussion

This article describes the co-adaptation of a school-based mental health literacy intervention for delivery in Vietnam and Cambodia to be tested in a cluster-randomised controlled feasibility trial. Through participatory workshops with diverse stakeholders, adaptations included changes to facilitation and content. Our approach was informed by co-creation in the AMP study, research team expertise, ADAPT guidance and de Boer *et al*’s recommendations for co-adaptation, enabling a systematic yet flexible approach.

Our findings suggest that, with careful planning and facilitation, co-adaptation can be implemented in LMIC settings where participatory approaches may be less common. A potential challenge in our study was stakeholder readiness for co-adaptation, which can vary with contextual power distance, that is, the degree to which power imbalances are accepted within a culture.^33^ Southeast Asian cultures often exhibit high power distance, reflecting greater acceptance of hierarchical social structures.^47^ We anticipated that adolescents may be hesitant to share their views in front of adults, including teachers and government representatives. Strategies such as involving adolescents in school leadership positions, separating adults and adolescents for small group work and using anonymous voting activities appeared to support active participation across all groups.^34^ That said, the presence of senior stakeholders may have influenced some participants’ responses, a limitation we could not fully mitigate. This suggests that co-adaptation using similar participatory approaches can be implemented in comparable contexts, provided strategies are designed with these dynamics and limitations in mind.

Implementing co-adaptation of a single intervention across four sites also introduced practical challenges. In Thái Bình, logistical constraints shortened the 3-day workshop to 1 day, requiring last-minute adjustments to activities and feedback sessions. Differences across sites in topic voting and workbook feedback meant that research team discussions were important to consider all stakeholder views and reach decisions on final adaptations. This highlights that co-adaptation requires flexibility, particularly when working across multiple contexts, and can be time- and resource-intensive. In M-BRIGHT, co-adaptation was an iterative process spanning early context analysis through SA and mapping, the workshops and an ongoing process evaluation to enable responsive adaptations. Additionally, the stakeholder advisory groups will have opportunities to feedback throughout delivery. For researchers working with more limited resources, pragmatism may be a key consideration; for example, prioritising resource-intensive activities such as in-person workshops in the contexts most different from the original intervention setting and using less intensive approaches such as online meetings for ongoing stakeholder engagement.

Beyond practical considerations, engaging stakeholders across multiple system levels (eg, interpersonal, organisational, community) was critical to our approach. Involving parents, teachers, community leaders, religious figures and policymakers alongside adolescents brought diverse perspectives and built buy-in for intervention delivery and sustainability. This is important given the influence of these groups on adolescents’ mental health beliefs and help-seeking behaviours. For example, in Cambodia, where mental disorders are often perceived as spiritual problems,^48^ help-seeking from religious figures is common. Additionally, policymakers play a key role in deciding how to prioritise adolescent mental health in national health agendas.^49^ During the workshop in Siem Reap, a Ministry of Education, Youth and Sport representative encouraged teachers to consider how the intervention could be sustained long-term. This highlights the value of multilevel stakeholder engagement in co-adaptation for embedding interventions in existing systems and fostering cross-sector collaboration from the outset.

This study also demonstrates the importance of equity in global health partnerships. Local research teams led on adapting the intervention with input from local stakeholders, enabling them to determine which components were most contextually relevant^50^ and strengthening local capacity within health and other systems (eg, education).^51^ This was a priority given the histories of European colonial rule in both countries, the lasting impacts of which on mental health system capacity and resource availability informed our capacity building and local adaptation approaches. By positioning local teams and stakeholders as decision-makers rather than recipients of interventions designed elsewhere, co-adaptation can help address historically embedded power imbalances in global health research.^52^ A practical challenge in achieving an equitable partnership was the difference among teams in prior knowledge of the AMP study. The Vietnam team’s involvement in AMP gave them familiarity with the co-creation process and core intervention components, while these were new to the Cambodia team. This was addressed through the study inception meeting, SA and mapping and South-South learning, with Vietnamese researchers supporting co-adaptation workshops in Cambodia. Researchers planning similar multi-country co-adaptation should allocate sufficient time and resources for knowledge transfer with researchers new to the original intervention.

Based on our experiences, we offer five practical takeaways for researchers planning co-adaptation: (1) engage diverse stakeholders from project inception and throughout the adaptation process; (2) facilitate participatory activities that enable all stakeholders, including intended end users, to share their perspectives; (3) conduct early context analysis, such as SA and mapping, to identify contextual similarities and differences prior to adaptation; (4) allow flexibility during workshop delivery and beyond, including enabling context-specific approaches if working across multiple sites and creating mechanisms to track and incorporate responsive adaptations; and (5) involve local research partners in decision-making to support contextual relevance and ownership of the process.

### Strengths and limitations

There are several strengths of our co-adaptation approach. First, we included early and continuous stakeholder engagement through our stakeholder advisory groups. Involving local communities and stakeholders helped ensure that the adapted intervention was sensitive to local needs, rather than assuming universal benefit.^52^ Second, we involved stakeholders across sectors, age groups and positions in the workshops. Third, we used participatory approaches in the workshops to reduce power imbalances. Finally, our research team included researchers involved in the AMP study and those new to M-BRIGHT, who were knowledgeable about the new study contexts.^34^

Several limitations should be acknowledged. First, adolescents and parents who participated in the co-adaptation workshops were identified through schools, which may have resulted in the under-representation of marginalised adolescents and families. These adolescents were also all in leadership positions at school. While this was done to ensure comfort in sharing their views, it may have skewed perspectives towards those who experience good mental health at school and feel positively about school-based programmes. That said, intervention participants represent a wide range of students, and their feedback will be obtained during intervention delivery and evaluation. Second, although steps were taken to minimise power imbalances, the presence of teachers and senior stakeholders may have influenced some participants’ willingness to express their views. Third, there was limited time available to collect feedback on all intervention topics from all participants. Instead, different groups were asked to comment on different topics, with at least two groups providing feedback on each topic. This also ensured that participants did not experience fatigue during participation in the workshops. Finally, the translation of workbook feedback may have resulted in some loss of nuance, despite careful review by members of the research team.

## Conclusion

Rather than a ‘one-size-fits-all’ model, mental health interventions require contextual adaptation to ensure acceptability and sustainability within existing health and other systems. We found that meaningful stakeholder engagement can be successfully completed in settings where there has been limited prior use of co-adaptation. Importantly, our findings suggest that such approaches can generate locally relevant adaptations while maintaining core intervention functions.

## Supplementary material

10.1136/bmjgh-2026-024547 online supplemental file 1

10.1136/bmjgh-2026-024547 online supplemental file 2

10.1136/bmjgh-2026-024547 online supplemental file 3

10.1136/bmjgh-2026-024547 online supplemental file 4

## Data Availability

Data are available upon reasonable request.
