# Supplementary material for: Co-adapting a school-based mental health literacy intervention for adolescents in Cambodia and Vietnam
Source: BMJ Glob Health. 2026 Sep 25;11(9):e024547. doi: 10.1136/bmjgh-2026-024547 (PMC13630099; doi:10.1136/bmjgh-2026-024547)
Supplement: Supplementary file 1 [file bmjgh-11-9-s001.docx]

| **Position** | **Site** | | | |
| --- | --- | --- | --- | --- |
|  | **Cambodia** | | **Vietnam** | |
|  | **Kampong Chhnang** | **Siem Reap** | **Thai Binh** | **Khanh Hoa** |
| **University academics** | - | - | 2 (1 M, 1 F) | - |
| **Government representatives** | 9 (4 M, 5 F) | 5 (4 M, 1 F) | 1 (F) | 2 (F) |
| **Civil society representatives** | 4 (1 M, 3 F) | 8 (1 M, 7 F) | - | - |
| **Parents** | 8 (3 M, 5 F) | 6 (F) | 5 (1 M, 4 F) | 8 (2 M, 6 F) |
| **Psychiatrists** | - |  | 2 (M) | 1 (M) |
| **Psychologists** | - | 1 (F) | - | 1 (M) |
| **Other health professionals** | - |  | 3 (1 M, 2 F) | 2 (1 M, 1 F) |
| **Religious figures (e.g. monks, pastors)** | 1 pastor (M) | 2 monks (M) | - | - |
| **Faith-based organisation representatives** | 1 (F) | 1 (M) | - | - |
| **School senior leadership** | 2 (1 M, 1 F) | 1 (F) | 2 (F) | 2 (1 M, 1 F) |
| **Adolescents** | 8 (3 M, 5 F) | 6 (1 M, 5 F) | 10 (5 M, 5 F) | 14 (3 M, 11 F) |
| **Teachers** | 2 (1 M, 1 F) | 5 (2 M, 3 F) | 4 (1 M, 3 F) | 5 (F) |
| **Youth Union representatives** | - | - | - | 3 (F) |
| **Total=** | **35 (14 M, 21 F)** | **35 (11 M, 24 F)** | **29 (11 M, 18 F)** | **38 (9 M, 31 F)** |

**Supplementary File 1.** Workshop participant details across the four study sites. ‘M’ and ‘F’ represent the numbers of males and females.
