# Supplementary material for: Co-adapting a school-based mental health literacy intervention for adolescents in Cambodia and Vietnam
Source: BMJ Glob Health. 2026 Sep 25;11(9):e024547. doi: 10.1136/bmjgh-2026-024547 (PMC13630099; doi:10.1136/bmjgh-2026-024547)
Supplement: Supplementary file 2 [file bmjgh-11-9-s002.docx]

**Supplementary File 2.** Overview of the co-adaptation workshop schedule.

**Day 1**

| **Time** | **Title / overview** | **Materials** | **Facilitated by** |
| --- | --- | --- | --- |
| 7:30-8:00 | Arrival of attendees |  |  |
| 8:00-8:05 | Opening remarks |  | Principal investigators (PIs)/Country leads |
| 8:05-8:10 | Speed Meeting   - How to play: Split the group into 2 lines facing each other (like speed dating). - Each pair has 1 minute to introduce themselves to each other (can be on a topic: hobbies, nicknames, a funny story…). - Then, 1 line moves to meet a new person. - After a few rounds, players share an interesting thing about a person they met. Fun because: Fast-paced, not boring, everyone gets to talk. | YouTube video of 30 second timer | Research assistants |
| 8:10-8:40 | Overview of the M-BRIGHT study, including phases and purpose of the co-adaptation workshops. | Presentation slides | PIs/Country leads |
| 8:40-9:55 | Overview of the AMP study intervention (the structure and topics of the intervention).  Introduction to the AMP study intervention topics: In groups, participants are given sheets of A1 paper with all topic names written on as well as cut-outs of topic descriptions. Participants are asked to glue the descriptions to the corresponding topics.  Groups feedback their responses. Answers are then presented, along with the objectives of the intervention topics, followed by the introduction of potential new topics that could be included in the M-BRIGHT intervention.  Intervention topic ranking activity:   - 15 A2 pieces of paper with the names of the topics stuck around the room. - Each participant given 9 (changed to 8 after first workshop) stickers or sticky notes. - Participants walk around the room and stick their stickers on the pieces of paper labelled with the topics they think are most important to include in the M-BRIGHT intervention. | Presentation slides  Information cards and A1 paper with topic names written on them  Presentation slides  A0 paper (cut into A2 size - 15 sheets of paper, each with a topic name written on it), stickers/sticky notes | Research assistants |
| 9:55-10:10 | Tea break |  |  |
| 10:10-11:30 | Presentation of the activities in the AMP study intervention, including how the indoor and outdoor sessions were run and some of the suggested changes that participants made to the intervention.  Participant feedback session on adapting intervention to new contexts: in groups, participants given large sheets of paper to note down what works, what won’t work and any suggestions on timings, locations, facilitation for the intervention. | Presentation slides  A1 sheets of paper, pens | CEI advisors/Research assistants |
| 11:30-13:30 | Lunch break |  |  |
| 13:30-13:45 | Ice breaker: following a dance to A Ram Sam Sam song | YouTube video |  |
| 13:45-15:15 | Facilitated small group work to review content of the AMP study intervention and suggest adaptations for M-BRIGHT:  In groups of adults and adolescents, participants given printouts of the AMP intervention manual (3 topics per adult group, 2 per adolescent pair - one copy each per group). Groups read through chapters and fill in workbook as a group (one workbook provided per topic).  For the new topics, groups asked to fill in the workbook with ideas for content and delivery (e.g. what students should learn, what activities should be included, what materials are needed). | Workbook, manual handouts, pens | Research assistants |
| 15:15-15:30 | Tea break |  |  |
| 15:30-16:30 | Groups feedback workbook responses to wider group and researchers facilitate discussion. |  | Country leads/Research assistants |

**Day 2**

| **Time** | **Title / overview** | **Materials** | **Facilitated by** |
| --- | --- | --- | --- |
| 7:30-8:00 | Arrival of participants |  |  |
| 8:00-8:15 | Ice breaker: Drawing emotions   1. Each participant given a sticky note and marker pen. 2. The following sentence written on A1 sheet: “How do you feel?” 3. Ask participants: “Please draw on your sticky note something that expresses how you feel right now (e.g., could be an animal, object etc.). 4. Ask participants to place their sticky notes on A1 sheet and whether anyone would like to describe their drawing. | Sticky notes, marker pens, A1 paper | Research assistants |
| 8:15-9:45 | Continuation of facilitated small group work to review content of the AMP study intervention and suggest adaptations for M-BRIGHT: | Workbook, manual handouts, pens | Research assistants |
| 9:45-10:00 | Tea break |  |  |
| 10:00-11:00 | Groups feedback workbook responses to wider group and researchers facilitate discussion. |  | County leads/Research assistants |
| 11:00-11:15 | Closing remarks. Reminder of tomorrow’s session. |  | PIs/Country leads |
| **Day 2 afternoon** | Research team meeting to debrief, collate and digitise workbook responses and prepare presentation of summary findings for tomorrow’s session. |  | Whole research team |

**Day 3**

| **Time** | **Title / overview** | **Materials** | **Facilitated by** |
| --- | --- | --- | --- |
| 7:30-8:00 | Arrival of participants |  |  |
| 8:00 -9:00 | Presentation on summary findings. | Presentation slides | County leads/Research assistants |
| 9:00 – 10:00 | Whole group discussion for participant to ask questions and offer additional feedback and suggestions. |  | County leads/Research assistants |
| 10:00-10:15 | Tea break |  |  |
| 10:15– 11:15 | Continuation of whole group discussion and opportunity for participants to feedback on experience of taking part in workshops. |  | County leads/Research assistants |
| 11:15-11:30 | Closing remarks |  | PIs/Country leads |
