# Supplementary material for: Co-adapting a school-based mental health literacy intervention for adolescents in Cambodia and Vietnam
Source: BMJ Glob Health. 2026 Sep 25;11(9):e024547. doi: 10.1136/bmjgh-2026-024547 (PMC13630099; doi:10.1136/bmjgh-2026-024547)
Supplement: Supplementary file 3 [file bmjgh-11-9-s003.docx]

| Topic | Number of votes | | | | |
| --- | --- | --- | --- | --- | --- |
|  | **Kampong Chhnang** | **Siem Reap** | **Khán Hòa** | **Thái Bình** | **Total** |
| Stress | 16 | 14 | 25 | 18 | 73 |
| Anxiety | 14 | 24 | 16 | 4 | 58 |
| Depression | 19 | 28 | 16 | 20 | 83 |
| Emotional regulation | 18 | 9 | 26 | 21 | 74 |
| Social relationships (with parents) | 23 | 25 | 24 | 22 | 94 |
| Social relationships (with peers) | 26 | 26 | 29 | 18 | 99 |
| School violence and bullying | 16 | 23 | 30 | 25 | 94 |
| Self-care | 25 | 25 | 20 | 14 | 84 |
| Behavioural disorders | 13 | 7 | 13 | 19 | 52 |
| Developmental disorders | 14 | 7 | 12 | 7 | 40 |
| Eating disorders and body image* | 19 | 4 | 22 | 9 | 54 |
| The Internet* | 27 | 20 | 29 | 22 | 98 |
| Self-harm and suicide* | 14 | 23 | 22 | 18 | 77 |
| Substance abuse | 19 | 6 | 21 | 14 | 60 |

**Supplementary File 3.** The results of the topic ranking activity across all co-adaptation workshops. The six new topics considered for inclusion in the intervention are highlighted in **bold**.

*****The new topics chosen for inclusion in the intervention.
