# Supplementary material for: Co-adapting a school-based mental health literacy intervention for adolescents in Cambodia and Vietnam
Source: BMJ Glob Health. 2026 Sep 25;11(9):e024547. doi: 10.1136/bmjgh-2026-024547 (PMC13630099; doi:10.1136/bmjgh-2026-024547)
Supplement: Supplementary file 4 [file bmjgh-11-9-s004.docx]

### BMJ Global Health Author Reflexivity Statement

Adapted from Morton, B., Vercueil, A., Masekela, R., Heinz, E., Reimer, L., Saleh, S., Kalinga, C., Seekles, M., Biccard, B., Chakaya, J., Abimbola, S., Obasi, A. and Oriyo, N. (2022), Consensus statement on measures to promote equitable authorship in the publication of research from international partnerships. Anaesthesia, 77: 264-276. <https://doi.org/10.1111/anae.15597>

| **Study conceptualisation** | |
| --- | --- |
| 1. How does this study address local research and policy priorities? | The M-BRIGHT study seeks to promote evidence-based training, enhance mental health literacy and support adolescent mental health. At the conclusion of an earlier study, AMP, which M-BRIGHT builds on, key elements of the current study were identified by AMP study participants and policy makers. Co-adaptation was also a key part of the M-BRIGHT process to ensure the intervention is locally relevant. Adolescent mental health is a priority in both countries owing to limited youth-focused formal services, stigma and low mental health literacy. The outcomes of this project will ultimately serve as a basis for advocating to mental health policymakers and increasing national budget allocation for mental health promotion at the community level, including through expanding the network of lay mental health workers and strengthening mental health systems in Vietnam and Cambodia. |
| 2. How were local researchers involved in study design? | The design of the co-adaptation was led by the Vietnam research team, particularly Vân Vũ who had been involved in co-creation in the AMP study. Members of the Cambodia research team adapted the co-adaptation process to the Cambodian context and delivered the workshops at the study sites. In both teams, Community engagement and inclusion (CEI) Advisors (including a young person who had been part of the AMP study) supported with workshop design and delivery. The UK research team played a supportive role throughout co-adaptation. |
| **Research management** | |
| 3. How has funding been used to support the local research team(s)? | The NIHR budget for this study was set up in such a way that the local research teams would have sufficient funding to carrying out all necessary activities. The local research teams also have autonomy over their own budgets to specify what is needed to carry out specific activities and to move funds across budget lines (provided they meet the donor regulations) to ensure that there is sufficient funds for all activities. Thus, the local research teams hold funds within their own budgets to support the training, salaries and resources needed for the local research teams to be able to carry out co-adaption alongside other M-BRIGHT study activities. |
| **Data acquisition and analysis** | |
| 4. How are research staff who conducted data collection acknowledged? | All research staff involved in the co-adaptation process (e.g. supporting and leading on workshop design and delivery) are co-authors on this paper. |
| 5. How have members of the research partnership been provided with access to study data? | All members of the research team across the UK, Vietnam and Cambodia have access to study data in a shared, secure online drive. The physical data from co-adaptation (e.g., workbooks, other paper materials) are held at the universities in Vietnam and Cambodia and were digitised by the local research teams for sharing across countries. |
| 6. How were data used to develop analytical skills within the partnership? | The ‘data’ gathered from the co-adaption workshops included completed workbooks, flip charts from the group work, notes about the processes and summaries and collation into excel spreadsheet of participants’ inputs. The process of collating and summarising, which involved a form of analysis/analytical skills, was carried out by the local research teams, with support from the UK team. The wider M-BRIGHT study also involves capacity building of local research teams on qualitative and quantitative analysis. |
| **Data interpretation** | |
| 7. How have research partners collaborated in interpreting study data? | The Vietnam and Cambodia teams led on summarising and collating the feedback from the co-adaptation workshops (with support from the UK team) in their respective countries. The local research teams then met to review findings and finalise content of the intervention, guided by the outcomes of the workshops. The first author then wrote up the process and outcomes of co-adaptation into this paper, with input from the local research teams. |
| **Drafting and revising for intellectual content** | |
| 8. How were research partners supported to develop writing skills? | Whilst the writing of this paper was led by an early career researcher (ECR) from the UK team, all co-authors were involved in reviewing and editing the paper. The M-BRIGHT study more broadly has funded two ECRs from the local research teams to attend a week-long writing/publication workshop with ongoing mentoring (to support the ECRs throughout the publication process). Additionally, many papers from the wider project will be led by the Vietnam and Cambodia teams once data from the intervention phase has been collected. Early and mid-career researchers will be supported through this process by the Country Leads and UK team. |
| 9. How will research products be shared to address local needs? | The main output of co-adaptation was the M-BRIGHT intervention manual tailored for delivery in Vietnam and Cambodia to support adolescent mental health and mental health literacy. M-BRIGHT uses a train-the-trainer model, meaning that the current intervention providers will be able to support new cohorts of providers in the future. The intervention manual, as well as the mental health literacy training manuals described in this paper, will also be made available freely online in local languages to support their use in wider contexts. |
| **Authorship** | |
| 10. How is the leadership, contribution and ownership of this work by LMIC researchers recognised within the authorship? | All researchers who were involved in this study in Vietnam and Cambodia are co-authors on this paper. This includes the co-Principal Investigator in Vietnam (Hoang-Minh Dang), Country leads in Cambodia (Bunna Phoeun, Sareth Khann) and early and mid-career researchers in both countries who were involved in design and delivery of co-adaptation in the study. |
| 11. How have early career researchers across the partnership been included within the authorship team? | Many of the co-authors on this paper are early career researchers, such as the first and second authors (a PhD student/research coordinator and intern), both CEI Advisors (undergraduate students) and several members of the Vietnam and Cambodia research team (PhD students, research assistants). |
| 12. How has gender balance been addressed within the authorship? | 10 of the 18 authors on this paper are women, reflecting the strong representation of women in the M-BRIGHT research team, including in leadership positions, project management roles and leads on co-adaptation. |
| **Training** | |
| 13. How has the project contributed to training of LMIC researchers? | Co-adaptation involved South-South learning as the Vietnam team shared their experiences of co-creation from the AMP study with the Cambodia team, who had less experience using co-creation/co-adaptation approaches. The Vietnam team therefore worked closely with the Cambodia team to design the co-adaptation workshops, and Vân Vũ supported with their delivery in Siem Reap. Following this, the Cambodia team then facilitated the workshops themselves in the other study site, Kampong Chhnang. Other members of the research team who had not been involved in delivery of co-creation in the AMP study, such as the CEI Advisors who are both new to research, also gained skills and experience in using these approaches. The broader M-BRIGHT study involves training local research teams on numerous research skills, including qualitative and quantitative data analysis; health economics and costing analyses; and policy and systems strengthening processes. |
| **Infrastructure** | |
| 14. How has the project contributed to improvements in local infrastructure? | A key component of M-BRIGHT (described in this paper) is the ongoing collaboration and input from the in-country stakeholder advisory groups. They meet regularly and took part in the co-adaptation workshops. One of their key roles is to explore how to embed the M-BRIGHT intervention into existing structures and institutions, especially schools. This process has already started; for example, as described in the paper, at one co-adaptation workshop in Cambodia, a representative from the Ministry of Education, Youth and Sport encouraged teachers to consider how the intervention could be sustained beyond the study period. This speaks to both strengthening systems and institutional capacity to sustain such interventions in the longer term. |
| **Governance** | |
| 15. What safeguarding procedures were used to protect local study participants and researchers? | Ethical clearance for the wider M-BRIGHT study was obtained from the UK university partner as well as from in-country institutional review boards. As part of the process, informed consent and/or assent were obtained from all participants, including those attending the co-adaptation workshops. This informed consent/assent included, ensuring anonymity, confidentiality, right to withdraw from the study at any time, safe data storage and information about relevant services should participants request them or feel distressed in any way. Additionally, the M-BRIGHT study team has developed safeguarding policies and codes of conduct for both Vietnam and Cambodia, drawing on existing policies in the UK and the countries. The in-country safeguarding policies ensure that there is good and timely identification of safeguarding issues, and a clear set of responsibilities, procedures and mechanisms for ensuring they are recorded, addressed, reported and managed properly. |
